# (Ca_*x*_Nd_11-*x*_)Ru_4_O_24_ (*x* = 4.175)

**DOI:** 10.1107/S1600536810046593

**Published:** 2010-11-17

**Authors:** Terutoshi Sakakura, Keita Okada, Hironaga Iguchi, Jun Wang, Nobuo Ishizawa

**Affiliations:** a Ceramics Research Laboratory, Nagoya Institute of Technology, Asahigaoka, Tajimi 507-0071, Japan

## Abstract

Single crystals of the title compound, calcium neodymium ruthenate, (Ca_*x*_Nd_11-*x*_)Ru_4_O_24_ (*x* = 4.175), have been grown by the flux method. The structure consists of two crystallographically independent RuO_6_ octa­hedra, which are isolated from each other and embedded in a matrix composed of the Ca and Nd atoms. There are seven *M* sites which accommodate the Ca and Nd atoms with different populations. Four *M* sites at general positions are enriched with Nd, whereas the remaining three *M* sites on twofold rotation axes are enriched with Ca. The coordination numbers of O atoms to the *M* sites range from 6 to 9. The mean oxidation state of Ru was estimated at +4.79 from the composition analysis. The title compound is non-centrosymmetric and potentially multiferroic.

## Related literature

For related compounds, see: non-centrosymmetric *I*4_1_ structure of Ca_11_Re_4_O_24_ (Jeitschko *et al.*, 1998 ▶); centrosymmetric *I*4_1_/*a* structures of Sr_11_Re_4_O_24_ (Bramnik *et al.*, 2000 ▶) and Ba_11_Os_4_O_24_ (Wakeshima & Hinatsu, 2005 ▶); centrosymmetric *I*2/*a* structure of Sr_11_Os_4_O_24_ (Tomaszewska & Müller-Buschbaum, 1993 ▶). For bond-valence sums, see: Adams (2001 ▶); Brown (1992 ▶).

## Experimental

### 

#### Crystal data

Ca_4.175_Nd_6.825_Ru_4_O_24_
*M* *_r_* = 1940.16
Tetragonal, 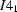

*a* = 11.2426 (2) Å
*c* = 16.1043 (3) Å
*V* = 2035.52 (6) Å^3^
*Z* = 4
Mo *K*α radiation
μ = 21.11 mm^−1^
*T* = 296 K
0.03 × 0.03 × 0.02 mm

#### Data collection

Bruker APEXII CCD diffractometer
Absorption correction: numerical (*SAINT*; Bruker, 2008 ▶) *T*_min_ = 0.507, *T*_max_ = 0.863
17423 measured reflections
5779 independent reflections
5453 reflections with *I* > 2σ(*I*)
*R*_int_ = 0.019

#### Refinement

*R*[*F* ^2^ > 2σ(*F* ^2^)] = 0.021
*wR*(*F* ^2^) = 0.047
*S* = 1.08
5779 reflections
125 parameters
Δρ_max_ = 3.05 e Å^−3^
Δρ_min_ = −1.86 e Å^−3^
Absolute structure: Flack (1983 ▶), 2535 Friedel pairs
Flack parameter: 0.44 (2)

### 

Data collection: *APEX2* (Bruker, 2008 ▶); cell refinement: *SAINT* (Bruker, 2008 ▶); data reduction: *SAINT*; program(s) used to solve structure: *SHELXS97* (Sheldrick, 2008 ▶); program(s) used to refine structure: *SHELXL97* (Sheldrick, 2008 ▶); molecular graphics: *ORTEPII* (Johnson, 1976 ▶); software used to prepare material for publication: *publCIF* (Westrip, 2010 ▶) and *PLATON* (Spek, 2009 ▶).

## Supplementary Material

Crystal structure: contains datablocks I, global. DOI: 10.1107/S1600536810046593/br2148sup1.cif

Structure factors: contains datablocks I. DOI: 10.1107/S1600536810046593/br2148Isup2.hkl

Additional supplementary materials:  crystallographic information; 3D view; checkCIF report

## Figures and Tables

**Fig. 1.**
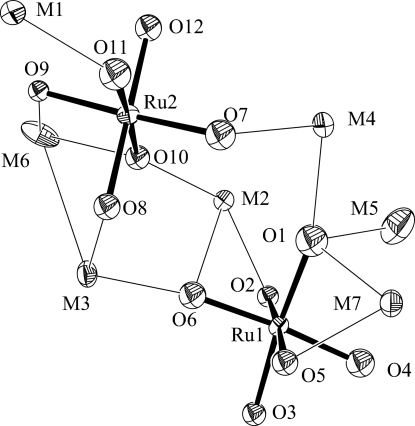
The asymmetric unit of M11Ru4O24 (M=Ca, Nd), showing the atom labelling and with displacement ellipsoids drawn at 95% probability level.

**Fig. 2.**
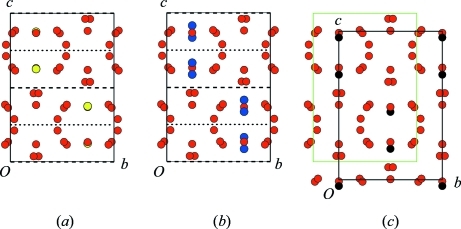
The M atom substructures in the I41/a non-split-atom model (a), the I41/a split-atom model (b) and the I41 model (c), projected along the a axis. The fully-occupied M atom site (yellow) in (a) becomes the split-atom site (blue) in (b), and turns into the fully-occupied one (black) in (c). All the other M atom sites (red) reside at similar positions in these substructures. Origin can be taken at any position along c in I41. The area corresponding to the I41/a unit cell is enclosed by green rectangle in (c).

## supplementary crystallographic information

### Comment

The structure of (Ca*_x_*Nd_11-_*_x_*)Ru_4_O_24_ (*x*=4.175) consists of two crystallographically independent RuO_6_ octahedra which are isolated from each other and embedded in a matrix composed of the Ca and Nd atoms, as shown in Fig. 1. The Ru1O_6_ octahedron is slightly distorted with a larger octahedral volume of 10.26 Å^3^ compared with Ru2O_6_ of 9.92 Å^3^. The composition analysis indicated that the mean oxidation state of Ru was +4.79, assuming formal charges for Ca, Nd and O.

If we assume that the oxidation state of Ru is 5+, the bond valence sums (BVSs) become 4.80 valence unit (vu) for Ru1 and 5.00 vu for Ru2 (Brown, 1992; Adams, 2001). If we assume that the oxidation state of Ru is 4+, BVSs become 4.05 vu for Ru1 and 4.21 vu for Ru2. Geometrical features of the Ru1O_6_ and Ru2O_6_ octahedra indicated that Ru^4+^ should enrich at Ru1. If we assume that the Ru1 site is occupied by Ru^4+^ and Ru^5+^, and that the Ru2 site is exclusively occupied by Ru^5+^, then the ratio of Ru^4+^:Ru^5+^ becomes 40:60 for Ru1 in the compound with *x*=4.175. The 40:60 ratio then leads BVS to 4.50 vu for the Ru1 site. This value is quite reasonable, suggesting that the crystal exhibits a partial charge disproportionation, i.e., Ru1 is occupied by Ru^4+^ and Ru^5+^ in almost even probabilities, whereas Ru2 is exclusively occupied by Ru^5+^. If Ru1 is occupied by Ru^4+^ and Ru^5+^ exactly in the equal proportion, then the mean oxidation state of Ru in the compound becomes +4.75, providing a commensurate composition Ca_4_Nd_7_Ru_4_O_24_ (i.e., *x*=4). The present crystal is very close to this ideal one.

There are seven *M* sites which accommodate the Ca and Nd atoms with different populations. The M1—M4 sites are located at the Wyckoff notation, 8*b* of *I*4_1_, whereas the M5—M7 sites are at 4*a*. The M1—M4 sites are enriched with Nd in contrast with the Ca-rich M5—M6 sites. The M7 site is almost exclusively occupied by Ca. Coordination numbers of O around the *M* site are 9 for M1 and M2, 8 for M3, M4, M6 and M7, and 6 for M5. The BVSs of Nd and Ca at all M sites were 3.0±0.2 and 2.0±0.2 vu, respectively, except for M6 where BVS of Nd was 2.55 vu, a slightly lower value than usual. Since the anisotropic ADP ellipsoid of M6 was relatively large and prolate, a possible small displacement of Nd from Ca could resolve the BVS problem.

The global instability indices, defined as the root mean square of the BVS deviation for all the atoms present in the asymmetric unit (Brown, 1992), were 0.14 and 0.17 vu for the oxidation states of 5+ and 4+ for Ru, respectively. These values lay within a modest deviation of ±0.2, suggesting the legitimacy of the present structure.

The present compound is isostructural with Ca_11_Re_4_O_24_ (*I*4_1_) (Jeitschko *et al.*, 1998) in which the mean oxidation state of Re is +6.5. In contrast with the present crystal, a complete charge disproportionation into +6 and +7 presumably occurs in Ca_11_Re_4_O_24_ over two crystallographically independent two Re sites from the geometrical consideration. On the other hand, several centrosymmetric structures were reported for Sr_11_Re_4_O_24_ (*I*4_1_/*a*) (Bramnik *et al.*, 2000), Ba_11_Os_4_O_24_ (*I*4_1_/*a*) (Wakeshima & Hinatsu, 2005) and Sr_11_Os_4_O_24_ (*I*2/*a*) (Tomaszewska & Müller-Buschbaum, 1993).

A difference in the tetragonal *I*4_1_ and *I*4_1_/*a* structures can be clearly seen in the substructure composed of M atoms, as shown in Fig. 2. The centrosymmetric tetragonal structures reported for Sr_11_Re_4_O_24_ (Bramnik *et al.*, 2000) and Ba_11_Os_4_O_24_ (Wakeshima & Hinatsu, 2005) are based on the *I*4_1_/*a* non-split-atom model containing 4 crystallographically independent M sites (Fig. 2a). This model was quite poor for the present crystal because one M site (coloured in yellow in Fig. 2a) at 4*b* in *I*4_1_/*a* showed an extraordinary prolate ADP ellipsoid along the *c* axis, as mentioned in the refinement section in detail. The *I*4_1_/*a* split-atom model, assuming 8*e* (blue in Fig. 2b) instead of 4*b*, was better than the *I*4_1_/*a* non-split-atom model, but still did not explain the observed weak reflections breaking the glide symmetries in *I*4_1_/*a*. The deviation of the M7 atom site (black in Fig. 2c) at 4*a* in *I*4_1_ from the corresponding one (yellow in Fig. 2a) in *I*4_1_/*a* is clear. Since M7 is virtually composed of Ca in the present crystal, its small ionic radius compared with Sr or Ba could be ascribed to the symmetry breaking into the noncentrosymmetric and polar structure. The presence of *I*4_1_/*a* structure in other compounds, however, may suggest a possible order-disorder transition of the present compound at elevated temperatures.

### Experimental

Powders of Nd_2_O_3_ (3 N, Wako chemical), RuO_2_ (3 N, Kojundo Chemical Laboratory Co. Ltd.) and CaCl_2_ (95.0%, Wako chemical) were mixed together with a mole fraction of 2:1:9 with a total weight of 4.97 g and put into an alumina crucible. The crucible was then placed on alumina powder in a larger alumina crucible. The double crucible was heated in air to 1373 K at the rate of 100 K/h, held for 10 h at 1373 K, cooled at the rate of 4 K/h to 973 K, and then furnace-cooled by turning off the power. The flux component was washed away by distilled water. Crystals were found in a block shape of 30–50 µm in diameter. Energy dispersive spectroscopy indicated that the Ca:Nd ratio was 4.1:6.9 with estimated uncertainty of ±0.3, which agreed with the ratio 4.175:6.825 obtained from the structure refinement.

### Refinement

A small monoclinic distortion of the body-centred tetragonal cell was reported on Sr_11_Os_4_O_24_ (Tomaszewska & Müller-Buschbaum, 1993). The unconstrained refinement of the unit-cell parameters in the integration procedure by *SAINT* (Bruker, 2008) on the present crystal, however, gave no significant deviation from the right angle.

Since the centrosymmetric space group *I*4_1_/*a* was reported for similar structures in the literature, the distinction between *I*4_1_ and *I*4_1_/*a* was examined on the present crystal. Systematic absence exceptions for the glide plane perpendicular to the tetragonal *c* axis amounted to 211 reflections in number, with the mean I/σ(I) being 2.5. The refinement assuming I4_1_/*a* with 65 parameters resulted in R1=0.066 for 3072 reflections with an extraordinarily prolate ADP ellipsoid along *c* for M4 at 4*b*. The residual electrons, 32 e Å^-3^ at 0.66 Å from M4 and -42 e Å^-3^ at 0.0 Å from M4, also indicated that M4 should be split. The refinement assuming a split atom model for M4 in *I*4_1_/*a* with 68 parameters resulted in R1=0.034, which still seemed significantly worse than 0.021 for the final *I*4_1_ model. The *I*4_1_/*a* model was thus discarded in the course of refinements. Because of significantly large displacements of M7 from the ideal position in the *I*4_1_/*a* non-split-atom model, PLATON (Spek, 2009) detected any additional symmetry neither for the M atom substructure nor for the full unit cell structure.

The refinement assuming the *I*4_1_ single domain structure resulted in R1=0.0212, S=1.077 and the Flack parameter *x*=0.44 (2). Another refinement assuming its enantiomer, which can be obtained by inverting the structure at the origin and subsequent shifting by *b*/2, resulted in R1=0.0214, S=1.082 and the Flack parameter *x*=0.47 (2). These results indicated that the crystal was composed of the two enantiomers with almost equal volumes.

Populations of Ca and Nd at seven *M* sites were refined with constraints to have no vacancies. The positional and atomic displacement parameters of Ca and Nd at each site were constrained to have the same values. The fractional coordinate *z* of Ru2 was fixed at 0.125 to define the origin along the *c* axis. The highest remaining peak was 1.33 Å from M7 and the deepest hole was 0.64 Å from M5.

### Crystal data

**Table:**

Ca_4.175_Nd_6.825_Ru_4_O_24_	*D*_x_ = 6.331 Mg m^−^^3^
*M**_r_* = 1940.16	Mo *K*α radiation, λ = 0.71073 Å
Tetragonal, *I*4_1_	Cell parameters from 7875 reflections
Hall symbol: I 4bw	θ = 2.2–40.0°
*a* = 11.2426 (2) Å	µ = 21.11 mm^−^^1^
*c* = 16.1043 (3) Å	*T* = 296 K
*V* = 2035.52 (6) Å^3^	Block, black
*Z* = 4	0.03 × 0.03 × 0.02 mm
*F*(000) = 3444	

### Data collection

**Table:**

Bruker APEXII CCD diffractometer	5779 independent reflections
Radiation source: fine-focus sealed tube	5453 reflections with *I* > 2σ(*I*)
graphite	*R*_int_ = 0.019
φ and ω scans	θ_max_ = 40.0°, θ_min_ = 2.2°
Absorption correction: numerical (*SAINT*; Bruker, 2008)	*h* = −19→20
*T*_min_ = 0.507, *T*_max_ = 0.863	*k* = −20→19
17423 measured reflections	*l* = −29→27

### Refinement

**Table:**

Refinement on *F*^2^	Secondary atom site location: difference Fourier map
Least-squares matrix: full	*w* = 1/[σ^2^(*F*_o_^2^) + (0.0127*P*)^2^ + 23.2349*P*] where *P* = (*F*_o_^2^ + 2*F*_c_^2^)/3
*R*[*F*^2^ > 2σ(*F*^2^)] = 0.021	(Δ/σ)_max_ = 0.002
*wR*(*F*^2^) = 0.047	Δρ_max_ = 3.05 e Å^−^^3^
*S* = 1.08	Δρ_min_ = −1.85 e Å^−^^3^
5779 reflections	Extinction correction: *SHELXL97* (Sheldrick, 2008), Fc^*^=kFc[1+0.001xFc^2^λ^3^/sin(2θ)]^-1/4^
125 parameters	Extinction coefficient: 0.000440 (12)
0 restraints	Absolute structure: Flack (1983), 2535 Friedel pairs
Primary atom site location: structure-invariant direct methods	Flack parameter: 0.44 (2)

### Special details

**Table:**

Geometry. All s.u.'s (except the s.u. in the dihedral angle between two l.s. planes) are estimated using the full covariance matrix. The cell s.u.'s are taken into account individually in the estimation of s.u.'s in distances, angles and torsion angles; correlations between s.u.'s in cell parameters are only used when they are defined by crystal symmetry. An approximate (isotropic) treatment of cell s.u.'s is used for estimating s.u.'s involving l.s. planes.
Refinement. Refinement of F^2^ against ALL reflections. The weighted R-factor wR and goodness of fit S are based on F^2^, conventional R-factors R are based on F, with F set to zero for negative F^2^. The threshold expression of F^2^ > 2σ(F^2^) is used only for calculating R-factors(gt) etc. and is not relevant to the choice of reflections for refinement. R-factors based on F^2^ are statistically about twice as large as those based on F, and R- factors based on ALL data will be even larger.

### Fractional atomic coordinates and isotropic or equivalent isotropic displacement parameters (Å^2^)

**Table:**

	*x*	*y*	*z*	*U*_iso_*/*U*_eq_	Occ. (<1)
Nd1	0.79795 (4)	0.73064 (4)	0.25967 (6)	0.00757 (9)	0.901 (3)
Nd2	0.20206 (4)	0.76978 (4)	−0.00960 (6)	0.00713 (8)	0.878 (3)
Nd3	0.29027 (5)	0.97654 (5)	0.16314 (7)	0.00811 (11)	0.599 (3)
Nd4	0.29060 (5)	0.47661 (5)	0.08656 (7)	0.00821 (11)	0.611 (3)
Nd5	0.0000	0.5000	0.00714 (9)	0.0188 (3)	0.422 (4)
Nd6	0.5000	1.0000	−0.00729 (9)	0.0158 (3)	0.379 (4)
Nd7	0.0000	0.5000	0.21035 (9)	0.0092 (2)	0.047 (3)
Ca1	0.79795 (4)	0.73064 (4)	0.25967 (6)	0.00757 (9)	0.099 (3)
Ca2	0.20206 (4)	0.76978 (4)	−0.00960 (6)	0.00713 (8)	0.122 (3)
Ca3	0.29027 (5)	0.97654 (5)	0.16314 (7)	0.00811 (11)	0.401 (3)
Ca4	0.29060 (5)	0.47661 (5)	0.08656 (7)	0.00821 (11)	0.389 (3)
Ca5	0.0000	0.5000	0.00714 (9)	0.0188 (3)	0.578 (4)
Ca6	0.5000	1.0000	−0.00729 (9)	0.0158 (3)	0.621 (4)
Ca7	0.0000	0.5000	0.21035 (9)	0.0092 (2)	0.953 (3)
Ru1	−0.00013 (6)	0.74968 (6)	0.12491 (7)	0.00535 (4)	
Ru2	0.49979 (6)	0.74990 (6)	0.1250	0.00552 (4)	
O1	0.1079 (4)	0.6131 (4)	0.0978 (3)	0.0138 (7)*	
O2	−0.0089 (4)	0.7999 (4)	0.0077 (3)	0.0065 (7)*	
O3	−0.1224 (3)	0.8782 (3)	0.1532 (2)	0.0079 (6)*	
O4	−0.1468 (4)	0.6608 (4)	0.1179 (3)	0.0121 (8)*	
O5	0.0110 (4)	0.7089 (4)	0.2448 (3)	0.0093 (8)*	
O6	0.1409 (4)	0.8488 (4)	0.1334 (3)	0.0106 (7)*	
O7	0.3972 (4)	0.6126 (4)	0.1538 (3)	0.0099 (7)*	
O8	0.4113 (4)	0.8283 (4)	0.2162 (3)	0.0086 (7)*	
O9	0.6105 (3)	0.8799 (3)	0.0988 (3)	0.0066 (6)*	
O10	0.3897 (3)	0.8277 (3)	0.0502 (3)	0.0079 (6)*	
O11	0.6052 (4)	0.6778 (4)	0.2061 (3)	0.0123 (8)*	
O12	0.5873 (4)	0.6714 (4)	0.0346 (4)	0.0094 (7)*	

### Atomic displacement parameters (Å^2^)

**Table:**

	*U*^11^	*U*^22^	*U*^33^	*U*^12^	*U*^13^	*U*^23^
Nd1	0.00648 (15)	0.00682 (15)	0.00943 (15)	−0.00027 (12)	0.00102 (12)	−0.00061 (12)
Nd2	0.00608 (15)	0.00676 (15)	0.00855 (15)	−0.00038 (12)	0.00078 (12)	−0.00054 (12)
Nd3	0.0085 (2)	0.00601 (19)	0.0098 (2)	−0.00132 (15)	−0.00053 (17)	0.00009 (14)
Nd4	0.00755 (19)	0.00627 (19)	0.0108 (2)	−0.00106 (14)	0.00007 (16)	−0.00121 (14)
Nd5	0.0125 (5)	0.0231 (6)	0.0207 (5)	−0.0049 (4)	0.000	0.000
Nd6	0.0196 (5)	0.0088 (4)	0.0189 (5)	0.0093 (4)	0.000	0.000
Nd7	0.0074 (3)	0.0067 (3)	0.0136 (4)	−0.0004 (3)	0.000	0.000
Ca1	0.00648 (15)	0.00682 (15)	0.00943 (15)	−0.00027 (12)	0.00102 (12)	−0.00061 (12)
Ca2	0.00608 (15)	0.00676 (15)	0.00855 (15)	−0.00038 (12)	0.00078 (12)	−0.00054 (12)
Ca3	0.0085 (2)	0.00601 (19)	0.0098 (2)	−0.00132 (15)	−0.00053 (17)	0.00009 (14)
Ca4	0.00755 (19)	0.00627 (19)	0.0108 (2)	−0.00106 (14)	0.00007 (16)	−0.00121 (14)
Ca5	0.0125 (5)	0.0231 (6)	0.0207 (5)	−0.0049 (4)	0.000	0.000
Ca6	0.0196 (5)	0.0088 (4)	0.0189 (5)	0.0093 (4)	0.000	0.000
Ca7	0.0074 (3)	0.0067 (3)	0.0136 (4)	−0.0004 (3)	0.000	0.000
Ru1	0.00461 (7)	0.00620 (7)	0.00523 (7)	−0.00037 (5)	−0.00005 (5)	0.00051 (5)
Ru2	0.00537 (7)	0.00597 (7)	0.00521 (7)	0.00064 (5)	−0.00009 (5)	0.00053 (5)

### Geometric parameters (Å, °)

**Table:**

Nd1—O11	2.406 (5)	Nd5—O1	2.285 (4)
Nd1—O5^i^	2.419 (5)	Nd5—O1^viii^	2.285 (4)
Nd1—O9^ii^	2.470 (4)	Nd5—O7^x^	2.389 (4)
Nd1—O4^i^	2.493 (5)	Nd5—O7^iv^	2.389 (4)
Nd1—O2^iii^	2.494 (4)	Nd5—O11^iv^	2.464 (4)
Nd1—O4^iii^	2.510 (5)	Nd5—O11^x^	2.464 (4)
Nd1—O12^ii^	2.525 (5)	Nd6—O3^iv^	2.418 (4)
Nd1—O3^i^	2.548 (4)	Nd6—O3^xi^	2.418 (4)
Nd1—O3^iii^	2.764 (4)	Nd6—O10^v^	2.479 (4)
Nd2—O10	2.408 (4)	Nd6—O10	2.479 (4)
Nd2—O2	2.412 (4)	Nd6—O9^v^	2.507 (4)
Nd2—O5^iv^	2.467 (5)	Nd6—O9	2.507 (4)
Nd2—O8^iv^	2.512 (5)	Nd6—O6^iv^	2.915 (4)
Nd2—O7^iv^	2.546 (4)	Nd6—O6^xi^	2.915 (4)
Nd2—O6	2.562 (5)	Nd7—O2^vi^	2.377 (4)
Nd2—O6^iv^	2.589 (4)	Nd7—O2^xii^	2.377 (4)
Nd2—O1	2.686 (4)	Nd7—O5^viii^	2.416 (4)
Nd2—O1^iv^	2.857 (4)	Nd7—O5	2.416 (4)
Nd3—O9^v^	2.219 (4)	Nd7—O1	2.524 (4)
Nd3—O6	2.262 (4)	Nd7—O1^viii^	2.524 (4)
Nd3—O8	2.316 (5)	Nd7—O4	2.865 (5)
Nd3—O12^vi^	2.358 (5)	Nd7—O4^viii^	2.865 (5)
Nd3—O3^vii^	2.501 (4)	Ru1—O4	1.931 (5)
Nd3—O10	2.713 (4)	Ru1—O6	1.942 (4)
Nd3—O10^vi^	2.753 (4)	Ru1—O2	1.973 (4)
Nd3—O5^iv^	2.864 (4)	Ru1—O5	1.989 (4)
Nd4—O7	2.225 (4)	Ru1—O1	2.005 (4)
Nd4—O4^viii^	2.292 (5)	Ru1—O3	2.046 (4)
Nd4—O12^ix^	2.314 (5)	Ru2—O10	1.936 (4)
Nd4—O8^iv^	2.350 (5)	Ru2—O11	1.942 (5)
Nd4—O1	2.571 (4)	Ru2—O12	1.966 (5)
Nd4—O11^iv^	2.620 (4)	Ru2—O9	1.965 (4)
Nd4—O11^ix^	2.845 (4)	Ru2—O7	1.981 (4)
Nd4—O2^vi^	2.943 (4)	Ru2—O8	1.981 (5)
			
O4—Ru1—O6	176.1 (2)	O10—Ru2—O11	176.1 (2)
O4—Ru1—O2	92.84 (19)	O10—Ru2—O12	93.5 (2)
O6—Ru1—O2	86.75 (18)	O11—Ru2—O12	90.3 (2)
O4—Ru1—O5	89.49 (19)	O10—Ru2—O9	86.29 (16)
O6—Ru1—O5	90.74 (19)	O11—Ru2—O9	93.92 (18)
O2—Ru1—O5	176.6 (2)	O12—Ru2—O9	81.85 (19)
O4—Ru1—O1	96.22 (18)	O10—Ru2—O7	97.22 (17)
O6—Ru1—O1	87.72 (17)	O11—Ru2—O7	82.67 (18)
O2—Ru1—O1	92.38 (18)	O12—Ru2—O7	96.60 (19)
O5—Ru1—O1	89.79 (18)	O9—Ru2—O7	176.3 (2)
O4—Ru1—O3	78.74 (16)	O10—Ru2—O8	86.56 (19)
O6—Ru1—O3	97.33 (16)	O11—Ru2—O8	89.6 (2)
O2—Ru1—O3	88.71 (16)	O12—Ru2—O8	179.7 (3)
O5—Ru1—O3	89.35 (17)	O9—Ru2—O8	98.42 (18)
O1—Ru1—O3	174.89 (18)	O7—Ru2—O8	83.13 (19)

Symmetry codes: (i) *x*+1, *y*, *z*; (ii) *y*, −*x*+3/2, *z*+1/4; (iii) *y*, −*x*+1/2, *z*+1/4; (iv) *y*−1/2, −*x*+1, *z*−1/4; (v) −*x*+1, −*y*+2, *z*; (vi) −*y*+1, *x*+1/2, *z*+1/4; (vii) −*x*, −*y*+2, *z*; (viii) −*x*, −*y*+1, *z*; (ix) −*x*+1, −*y*+1, *z*; (x) −*y*+1/2, *x*, *z*−1/4; (xi) −*y*+3/2, *x*+1, *z*−1/4; (xii) *y*−1, −*x*+1/2, *z*+1/4.
